# Detection of Different Patterns of Genome-Wide Gene Expression Disturbance in Three Nullisomy Lines in Allotetraploid *Brassica napus*

**DOI:** 10.3390/plants14101434

**Published:** 2025-05-10

**Authors:** Shaolin Lei, Bo Wei, Qi Hu, Lang Liu, Feng Yu, Tuo Zeng, Xuye Du, Lei Gu, Hongcheng Wang

**Affiliations:** 1 School of Life Sciences, Guizhou Normal University, Guiyang 550025, China; leisl66@126.com (S.L.); 232100100408@gznu.edu.cn (B.W.); 232200101543@gznu.edu.cn (Q.H.); 202409006@gznu.edu.cn (L.L.); 201711004@gznu.edu.cn (F.Y.); zengtuo@gznu.edu.cn (T.Z.); duxuye@gznu.edu.cn (X.D.); 2 Guizhou Oil Crops Research Institute, Guizhou Academy of Agricultural Sciences, Guiyang 550006, China; 3 National Key Laboratory for Germplasm Innovation & Utilization of Horticultural Crops, College of Horticulture & Forestry Sciences, Huazhong Agricultural University, Wuhan 430070, China

**Keywords:** *Brassica napus*, chromosome loss, nullisomy, transcriptome

## Abstract

Aneuploidy-related disruptions are generally tolerated in polyploid plants, which exhibit a greater capacity for genomic compensation. In this study, we utilize allotetraploid *Brassica napus* as a model and generated three aneuploid variants (NC1, NC2, and NC8) to investigate the phenotypic and transcriptional consequences of chromosome loss. Significant phenotypic variations were observed, with the most notable being a marked dwarfing phenotype in the aneuploid materials compared to the euploid Oro. Transcriptomic analysis revealed widespread alterations in gene expression across the entire genome in the deficient variants. Notably, most of the differentially expressed genes (DEGs) were attributed to trans-acting effects resulting from the deletion of C chromosomes. Deletion of the C chromosomes induced gene expression changes not only on the corresponding chromosomes, but also on the affected genes across other chromosomes. Specifically, in the C1-deleted variant, the average gene expression of the A1 chromosome increased, while the number of expressed genes on other chromosomes decreased. In contrast, for C2 and C8 deletions, the average expression levels of homologous genes decreased, but the number of expressed genes on other chromosomes increased. These findings shed light on the complex compensatory mechanisms that underlie aneuploidy in polyploid plants and provide valuable insights into how plants maintain genomic stability despite chromosomal loss.

## 1. Introduction

Non-polyploidy is a phenomenon characterized by the presence of one or more normal numbers (2n) of extra chromosomes or the absence of chromosomes in a cell, resulting in an imbalance of gene dosages in the species. The balance of gene dosages is essential for the proper functioning of biological systems, and this imbalance can cause severe performance syndromes both in animals and plants [1,2]. While plants generally display greater tolerance to aneuploid than animals, since they have a stronger resistance to the lethality caused by chromosome deletion and show vitality [2,3], they still exhibit various phenotypic syndromes, such as delayed development, partial sterility, and morphological changes. There are significant differences in the ability of various plant species, as well as different variants within the same plant species, to tolerate gene dosage imbalances caused by aneuploidy. Each chromosome or chromosome pair plays a certain role in the growth and development of animals and plants by exhibiting phenotypic and growth abnormalities and defects [4,5,6]. However, these phenotypic abnormalities often vary significantly among different species, even for the deletion or addition of different chromosomes. For most animals and plants, losing a copy of a specific homologous chromosome is often fatal. However, some plants exhibit different phenotypes due to the presence of a whole set of trisomy with an additional copy of each chromosome pair. In the heterozygous allohexaploid common wheat (*Triticum aestivum* L.), the relevant genomes in allopolyploid species from different ancestors achieve functional mutual compensation [7]. Due to the strict chemical dose disruption of all dose-sensitive gene products encoded by chromosomes or chromosome subgenomes, the non-diploid regulation of gene expression by the entire chromosome or chromosome fragment in the species is more severe [8,9,10]. This indicates that polyploidy is a continuous process until a stable polyploid state is attained, and maintaining the number of chromosomes at or near the diploid level may be necessary for dose balance [11,12,13]. The gene dose imbalance in aneuploidy is usually regulated by the gene dose compensation mechanism to maintain the stability of gene expression and avoid adverse consequences, such as overexpression or gene silencing. The deletion or addition of chromosomes often results in changes in gene expression. Using RNA-seq technology and gene expression analysis, it can be found that the expression of specific genes changed or the number of genes increased due to changes in different chromosomes. Genetic studies of aneuploids have greatly advanced our early understanding of gene structures and the homologous relationships between different genomes in allopolyploids.

Nullisomy is a form of chromosomal numerical abnormality characterized by the complete loss of a pair of chromosomes in an individual or cell. This phenomenon represents a manifestation of aneuploidy, which is prevalent across plants and microorganisms [14,15,16]. The selection of genes with excellent traits can be expedited by artificially inducing the loss of chromosomes or segments [17]. For example, in genetic research on common wheat, the function of genes located on specific chromosomes can be investigated by creating targeted chromosome defects such as 2D defects [18,19]. Similarly, genes related to drought tolerance and disease resistance in rice have been rapidly identified through the construction of specific chromosome deletions. Furthermore, studies on chromosomal abnormalities have provided new insights into evolutionary biology. The reshaping of polyploid plant genomes and the phenomenon of chromosome loss indicate that chromosomal abnormalities are not only genetic problems at the individual level, but, rather, they may serve as significant driving forces for species evolution. For example, research on the artificial synthesis of rapeseed has shown that chromosome loss and rearrangement in early generations promote the formation of genome stability, thereby providing a model for understanding the origin of polyploid species [20]. These examples underscore the critical role of chromosomal abnormalities in regulating adaptability and gene expression patterns. Allopolyploid *Brassica napus* L. (2n = 38, AACC) is one of the most important oil crops globally, originating from two diploids *B. rpapa* L. (2n = 20, AA) and *B. oleracea* L. (2n = 18, CC) approximately 7500 years ago [21]. This genome structure contributes to the high genetic diversity of *Brassica napus*, which is significant for its adaptation to various environments and its application in diverse agricultural systems. *Brassica napus* L. has been used as a model system for studying genomic changes and interactions during allopolyploidy [7,22,23]. Studies have demonstrated that there are non-random changes in gene expression between the A and C genomes in the newly synthesized allotetraploid (AACC) of *Brassica napus* L., driven by intergenomic interactions, chromatin remodeling, and epigenetic mechanisms such as DNA methylation and histone modification [24]. It is reported that the gene expression of disequilibrium model not only affects the morphology and growth of plants, but also regulates plants’ adaptability and resistance properties, even the economical traits [24,25]. Regarding genome structure, *Brassica napus* experienced gene rearrangement and chromosome interaction, which played important roles in maintaining genome balance [12]. Additionally, changes in gene expression extend beyond gene recombination to include significant changes in alternative splicing. Variations in alternative splicing patterns may be an important factor affecting phenotypic diversity, growth, development, and lipid synthesis through the modulation of gene functions [23,26]. Meanwhile, the evolution of the rape genome after the Neolithic Age authenticated the role of gene recombination, duplication, and the formation of new genes in driving phenotypic changes and the adaptive evolution of rape [27].

In this study, we screened the offsprings from the cross and backcross between *Brassica napus* and the previously obtained restituted *B. rapa* (RBR, 2n = 20, AnAn) [7], and identified three nullisomy lines of C1 (NC1), C2 (NC2), and C8 (NC8) in natural *Brassica napus* for the first time. These three nullisomy lines were compared to Oro (Ctr) to reveal the gene expression patterns of plants with C chromosome deletion. Consequently, the phenotypical and transcriptional consequences of chromosome loss were investigated in order to reveal the response pattern of the nullisomy plants in response to C chromosome deficiency.

## 2. Results

### 2.1. FISH Analysis of Brassica napus Nullisomy and Euploid Oro Lines

**Figure A1:**
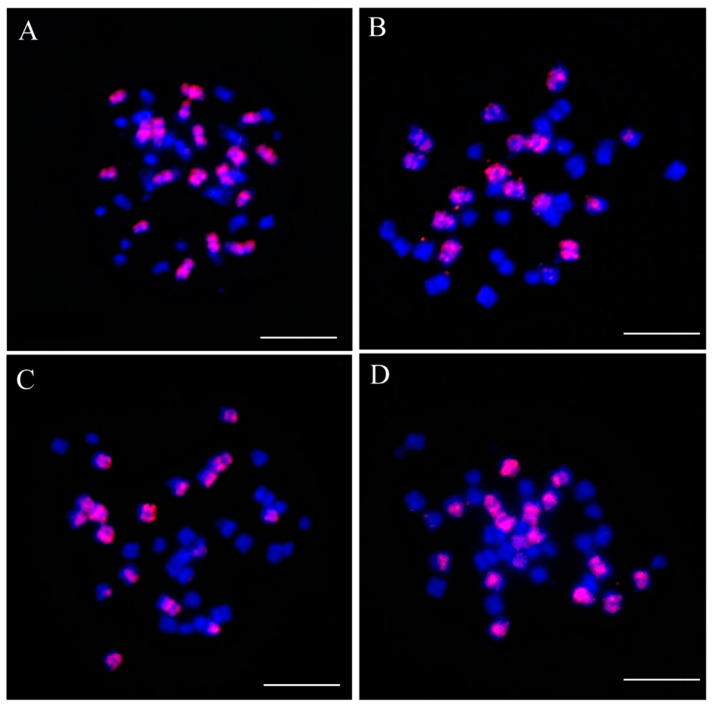
FISH analyses of three deficient and euploid Oro cells. Blue signals are DAPI, red signals are BAC BoB014O06 probe signal (biotinylated). (**A**) euploid Oro somatic cells with 38 chromosomes (blue), including 18 C subgenome chromosomes (red); (**B**–**D**) NC1, NC2, and NC8 somatic cells with 36 chromosomes (blue), including 16 C subgenome chromosomes (red). Bar = 50 μm.

**Figure A2:**
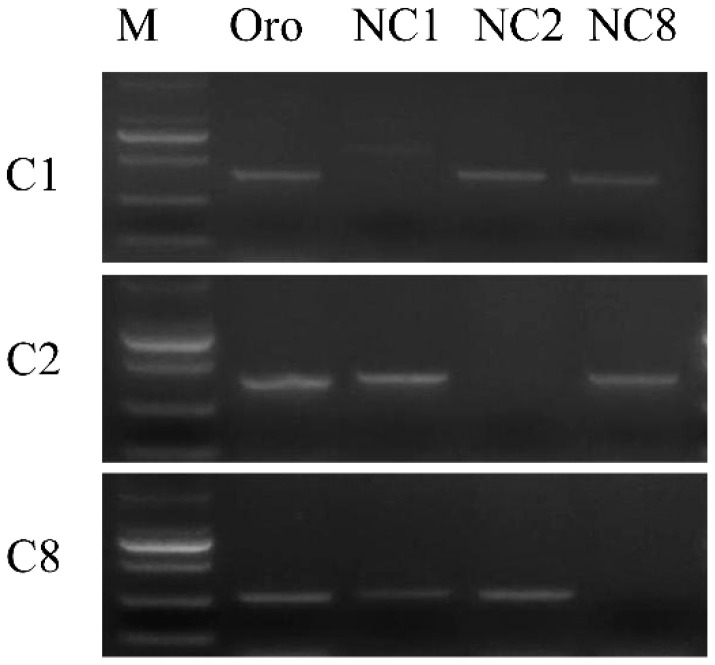
Identification of three nullisomy plants in *Brassica napus* using molecular markers. M represents Maker, Oro for parents, NC1 represent plants missing a pair of C1 chromosomes, NC2 represents plants missing a pair of C2, and NC8 represent plants missing a pair of C8.

To determine the number of missing chromosomes in *B. napus*, we applied FISH and C genome-specific probes to the three nullisomy (NC1, NC2, and NC8) and Oro lines. The FISH results show that all three nullisomy lines had lost only one pair of C chromosomes (Figure A1), since only 16 C chromosomes were seen in the nullisomic lines compared to the 18 C chromosomes seen in the euploid Oro control. To validate specific deletion staining, we identified the three deleted chromosomes with molecular markers. The results show that the three missing chromosome pairs were C1, C2, and C8 in the NC1, NC2, and NC8 lines, respectively (Figure A2). Subsequently, we selected the leaves of three missing bodies (NC1, NC2, and NC8) and Oro with consistent growth. Each genotype included three biological replicates, totaling twelve samples, each with a mass of 0.1–0.2 g to satisfy the transcriptome sequencing requirements, for subsequent analysis.

### 2.2. The Morphology of Nullisomy Plants

**Figure 1:**
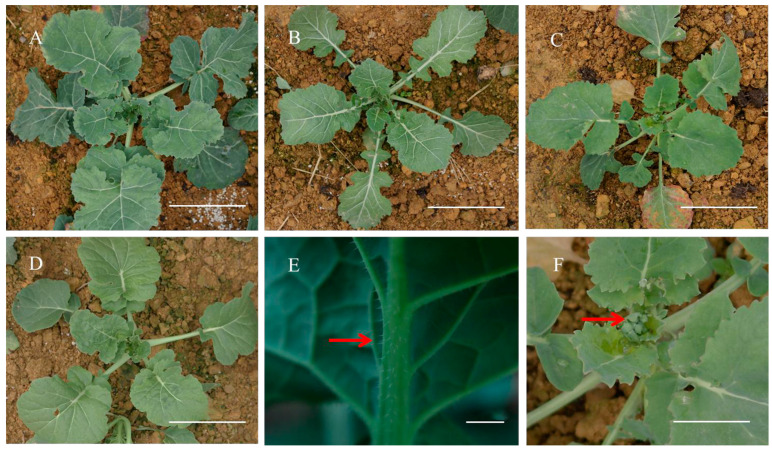
The phenotypes of aneuploid (NC1, NC2, NC8) and euploid Oro at a seedling stage of 48 days. (**A**) Euploid Oro seedling; (**B**) NC1 seedling; (**C**) NC2 seedling; (**D**) NC8 seedling; (**E**) burrs observed on the back side of NC1 seedlings; (**F**) buds observed in NC2 at seedling stage. (**A**–**D**,**F**) Bar = 10 cm, (**E**) Bar = 1 cm.

**Figure 2:**
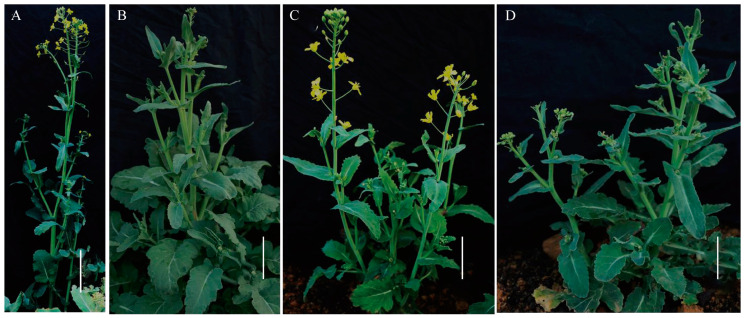
The performance of flowering time of euploid Oro and aneuploid (NC1, NC2, NC8) at 120 days. (**A**) Euploid Oro; (**B**) NC1; (**C**) NC2; (**D**) NC8. (**A**), Bar = 20 cm; (**B**–**D**), Bar = 10 cm.

We documented the phenotypes of NC1, NC2, and NC8 strains during both the seedling and flowering stages (Figure 1 and Figure 2). In the seedling stage, the leaves of these nullisomy individuals were smaller were shorter compared to the euploid Oro (leaf size Oro = 13.45 ± 0.32 cm, NC1 = 10.22 ± 0.32 cm, NC2 = 9.86 ± 0.39 cm, NC8 = 9.72 ± 0.30 cm, ANOVA *p* < 0.01, petiole length Oro = 9.55 ± 0.24 cm, NC1 = 5.72 ± 0.37 cm, NC2 = 5.49 ± 0.23 cm, NC8 = 5.68 ± 0.41 cm ANOVA *p* < 0.01). Additionally, the Oro seedlings exhibited a darker green color than the three nullisomy plants. Notably, burrs were observed undersides of the leaves in NC1, indicating the presence of genes related to burr development in chromosome C1 (Figure 1E). During the same period, a few buds were noted in NC2 (Figure 1F). Subsequently, we captured the flowering phenotypes of the nullisomy lines (Figure 2) and found that NC2 flowers approximately two months earlier than Oro, and the flowering time lasts longer (NC2, 65 days; Oro, 42 days). Moreover, NC2 plants were significantly shorter in height than Oro plants (Oro = 130.6 ± 2.8 cm, NC1 = 123.3 ± 2.4 cm, NC2 = 72.8 ± 2.4 cm, NC8 = 62.5 ± 3.0 cm) (Figure 2B). Furthermore, NC8 plants displayed a greater number of branches and shorter heights compared to Oro plants (Figure 2C). These observations indicate that the phenotypic characteristics of the C chromosome vary in multiple aspects following deletion.

### 2.3. RNA-Seq Reveals the Deletion of Chromosomes C1, C2, and C8

**Figure 3:**
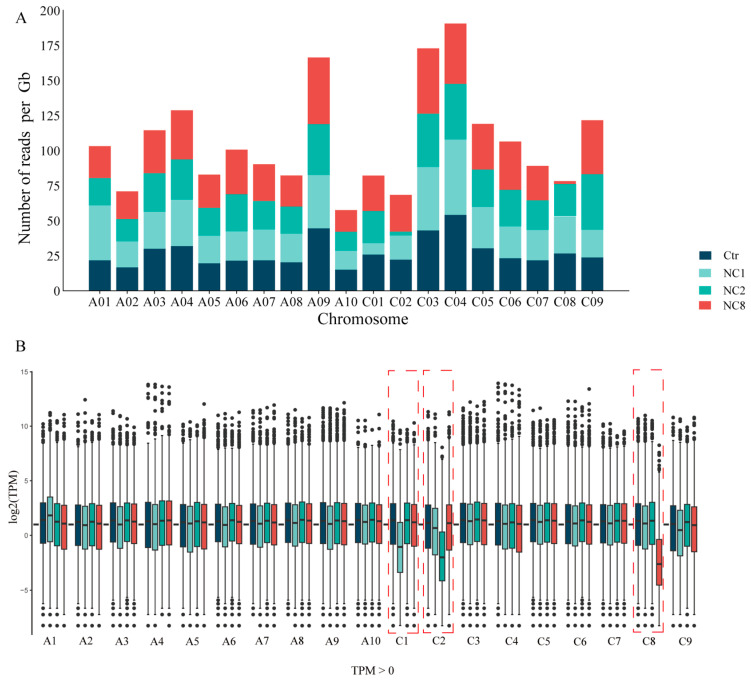
(**A**) Accumulation of readings of euploid Oro and NC1, 2, and 8 nullisomy lines. (**B**) Box plots of all expressed genes (TPMs) along all chromosomes.

To determine the identity of the deletion patterns and the perturbation of the genome-wide gene expression following chromosomes deletion, we compared nullisomy plants with euploidy controls using RNA-seq. The clean reads obtained from the raw data of each sample were aligned with the reference genome after filtering out Poly-N and removing low-quality reads. The TPM values, which were calculated using RSEM with default parameters (transcripts Per Kilobase of exon model per Million mapped reads), were used to determine gene expression levels. We then calculated the number of reads per gigabyte across all chromosomes (Figure 3A) and found that, in comparison to euploidy controls, the observed changes for each deletion chromosome were 0.135, 0.122, and 0.086, respectively. Subsequently, boxplots of all expressed genes (TPM > 0) were used to verify their respective expression trends (Figure 3B). An analysis of specific gene expression from each chromosome indicates that hypochromatosis of the nullisomy lines samples are C1, C2, and C8, respectively.

### 2.4. Overall Differential Expression of Genes Between the Aneuploid and Euploid

**Figure 4:**
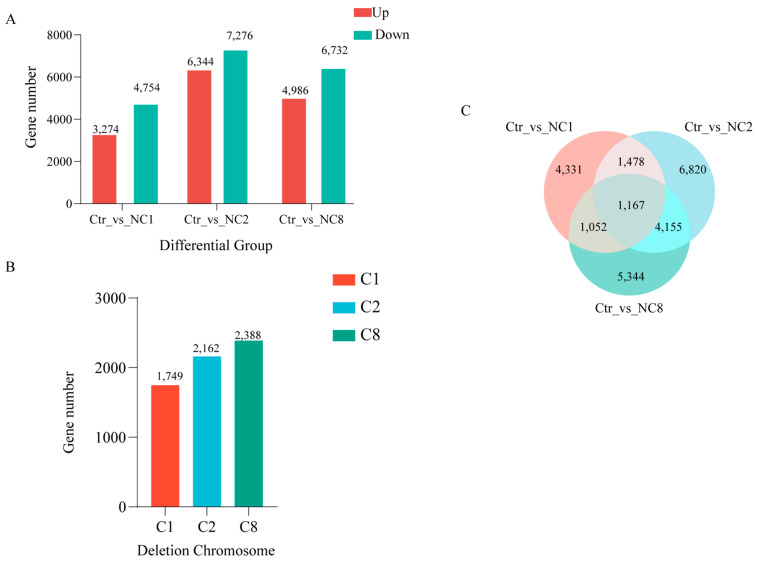
DEGs between euploid Oro and aneuploidy. (**A**) The number of DEGs of NC1, NC2, NC8, and euploid Oro. (**B**) The number of DEGs between the Oro and the nullisomy lines attributed to the corresponding C chromosome deletions. (**C**) Venn diagram comparing the differential number of genes expressed that result directly from the deletion of the C chromosome.

To evaluate the effect of chromosome deletion on gene expression, differentially expressed genes (DEGs) were screened using DESeq2 (FDR < 0.05 and |log2FC| ≥ 1). Fold Change reflects the fold change in gene expression between the experimental group and the control group. It is a simple and effective way to measure gene expression differences. A total of 8028, 13,620, and 11,718 DEGs were detected in NC1, 2, and 8, respectively, by comparing this three lines to the euploid Oro line. The upregulated DEGs comprised 3274 (40.78%), 6344 (46.58%), and 4986 (42.55%) in the NC1, NC2, and NC8 lines, respectively (Figure 4A). With the exception of NC2 (χ^2^, *p* < 0.01), no significant differences were observed between the upregulated and downregulated DEGs (χ^2^, *p* > 0.05). In total, 1749 (21.79%) DEGs between Oro and NC1 were directly attributed to the C1 chromosome deletion, while 2162 (15.87%) DEGs in Oro vs. NC2 were directly caused by the C2 deletion chromosome (Figure 4B). We also found that 2388 (20.38%) DEGs between Oro and NC8 were directly caused by C8 deletion (Figure 4B). This suggests that most of the DEGs have a dominant trans-acting effect (Figure 4B). Trans-acting refers to changes in gene expression that affect other chromosomes after the number of chromosomes is changed, which is indirectly caused by regulatory factors such as transcription factors, non-coding RNA, and protein complexes. The Venn diagram analysis across aneuploid and euploid lines showed that a total of 1167 DEGs were identified in the three nullisomic lines deficient in C chromosomes (Figure 4C). These results suggest the presence of specific DEGs associated with C chromosome deletion in *Brassica napus*, which is crucial for future studies on the expression patterns of other C chromosome deletion-related genes. We then counted the number of DEGs at different multiples of difference to reveal how the rest of the genome responds to the deletion of C chromosomes (C1, C2, and C8). We calculated three sets of differential genes at different multiples. In Ctr vs. NC1 and Ctr vs. NC2, the number of upregulated genes was slightly higher than that of downregulated genes, but in Ctr vs. NC8, the number of downregulated genes was gradually more than upregulated genes, with an increase in multiples (Table 1).

### 2.5. The Effect of Hypochromatosis on the Gene Expression of the Other Chromosomes of the Plant Genome

To assess whether the remaining chromosomes in aneuploidy lines exhibit a random contribution of DEGs, we calculated the genome-wide expressed genes of the remaining chromosomes and the proportion of DEGs between two distinct pairs. Specifically, we employed the ratio of DEGs to expressed genes (RDEGs/EGs) and the ratio of expressed genes to reference genes (REGs/RGs) to evaluate the differences among chromosomes (Table 2, Table A2 and Table A3). The analysis of expressed genes was restricted to those with a threshold (TPM > 0) across all three biological replicates, comparing euploid with aneuploid lines. It is worth noting that, in the comparisons of Ctr vs. NC1 (Table 2), Ctr vs. NC2 (Table A2), and Ctr vs. NC8 (Table A3), the proportions of expressed genes along the corresponding deleted C chromosomes were 29.20%, 19.03%, and 20.43%, respectively, which were significantly lower than those of the remaining chromosomes (Table 2) (P_NC1_ = 1.3379 × 10^−^^10^; P_NC2_ = 6.4785 × 10^−^^12^; P_NC8_ = 8.8186 × 10^−^^12^). The ranges of the proportion of expressed genes along the chromosomes in Ctr vs. NC1 (Table 2), Ctr vs. NC2 (Table A2), and Ctr vs. NC8 (Table A3) are 29.20–57.86%, 19.03–58.33%, and 20.43–60.24%, respectively.

Subsequently, the R_DEGs/EGs_ of the remaining chromosomes were evaluated to reveal whether the hypochromatosis has a contribution to preferential expression or repression. In the comparison of Ctr vs. NC1, the lowest R_DEGs/EGs_ was observed at 5.14% in C08 and the highest 35.06% in A1, with little difference among chromosomes of the high, middle, or low groups. Similarly, we counted R_(DEGs/EGs)_ in Ctr vs. NC2 and Ctr vs. NC8. The results show that the lowest A10 (6.92%) and highest A05 (24.41%) were found in Ctr vs. NC2 and the lowest A10 (10.66%) and that the highest A05 (24.60%) were found in Ctr vs. NC8. In order to determine whether the remaining chromosomes are sensitive to aneuploidy, we categorized them into three groups: low (the first five), middle 8 (the middle eight), and high (the last five). Consequently, A01, C02, and A02 are classified as high groups in NC1 (Table 2). In NC2 (Table A2), A05, A04, and C04 are the high groups, while in NC8 (Table A3), the high-sensitivity groups consist of A05, A04, and A02. However, we noticed that the value of R _(DEGs/EGs)_ of A01 in NC1 was more than twice that of the next highest group. Therefore, we re-evaluated EGs and DEGs on each chromosome in NC1. We found that, due to the loss of C01, the number of DEGs on A01 was the highest. Compared to the other two groups (Ctr vs. NC2 and Ctr vs. NC8 the number of DEGs on A02 (Table A2, 338) or A08 (Table A3, 450) in A01 (Table 2, 824) was about twice that of the two, but there was no such difference in the EGs on the chromosome. This might be because the deletion of C1 in NC1 is compensated by an increase in the gene expression levels on A01, while NC2 and NC8 compensate by increasing the number of gene expressions on the remaining chromosomes. This might be the direct cause of the significant differences within the same group, and this also implies that the compensation mechanism existing in NC1 is different from that in NC2 and NC8. This indicates that these chromosomes exhibit different sensitivities to aneuploidy, and this also indicates the differential resistance of different chromosomes to aneuploidy.

### 2.6. Perturbation of Gene Expression Due to Chromosome Deletion

**Figure 5:**
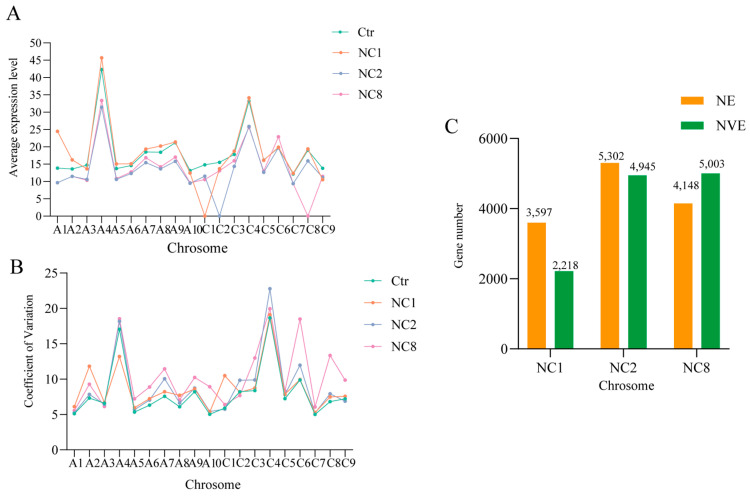
Changes in gene expression after the deletion of the C chromosome. (**A**) The average gene expression level on the remaining chromosomes (2n = 36) after chromosome deletions. (**B**) Coefficient of variation (COV) in each chromosome. (**C**) The number of newly expressed genes and no-expression genes appeared after deletion of C1, C2, and C8. NVE represents the number of newly expressed genes after chromosome deletion. NE indicates the number of no-expression genes after chromosome deletion.

In order to clarify the effect of chromosome deletion on global genome expression, we compared the expression levels of all expressed genes (TPM > 0) across the remaining chromosomes with an arithmetic average. Notably, after the deletion of C1, C2, and C8, high mean expression and Oro were observed in A1, A2, and A8 (Figure 5A) (Student’s *t* test, *p* < 0.5). This phenomenon may be attributed to the gene disturbance caused by chromosome deletion, which appear to influence the expression of the remaining chromosomes. To assess the dynamic changes in gene expression between euploidy and the three aneuploidies, we analyzed the coefficient of variation (COV) of each chromosome. By comparing the coefficient of variation for each chromosome, we can assess the impact of C chromosome deletion on gene expression across the remaining chromosomes. A higher coefficient of variation indicates a greater influence of C chromosome loss on that chromosome. The results show that COV was the highest among the three aneuploidies, with A4 in NC1, A4, C9 in NC2, and A3 in NC8 exhibiting slightly lower values (Figure 5B). In addition, we noticed that, after the loss of the C chromosome in the three groups, except for the loss of chromosomal chromosomes and homologous chromosomes, the coefficient of variation in A5, A6, A7, A9, A10, C3, C5, and C7 all increased due to the loss of chromosomes. This might imply that after the loss of the C chromosome in *Brassica napus*, all these chromosomes will have the possibility of an increased coefficient of variation. When comparing the expression changes in the four types of genes, COV was the highest in all three aneuploidy. In the comparison of the four groups, C4 and A4 showed the highest COV, while A10 and C7 showed the lowest COV. This indicates that C4 and A4 have higher variations compared to other chromosomes, that is, they have greater dynamic changes in gene expression. Conversely, A10 and C7 exhibit lower variations, smaller dynamic changes in gene expression, and more stable expression levels. Subsequently, we found that genes that were not expressed by Oro appeared in the aneuploid (Figure 5C), and, conversely, genes that were expressed in Oro but were not expressed in the aneuploid lines. We removed these genes and observed that 2218, 4945, and 5003 genes were newly expressed in Ctr vs. NC1 Ctr vs. NC2 and Ctr vs. NC8, respectively (Figure 5C). In contrast, the absence of 3597, 5302, and 4148 genes were observed in the three groups, respectively, indicating that the expression of some genes may be increased in response to the deletion of different C chromosomes and that these genes may either reside on the missed C chromosomes or directly regulated by the deletion of genes. For the three aneuploidy NC1, NC2, and NC8 we used; due to the deletion of a pair of C chromosomes, we calculated that the gene dose of their missing chromosomes was from 1 to 0.5 (2/4). To this end, we compared the newly expressed genes caused by the deletion of C chromosome with the dose–response model [1,2]. By comparing the FC (Fold Change) of the three groups, we found that more than 50% (1318), 60% (3411), and 70% (3596) of 2218, 4945, and 5003 newly expressed genes found in the three groups, namely Ctr vs. NC1, Ctr vs. NC2, and Ctr vs. NC8, were overcompensated (FC > 1). Therefore, there is a general overcompensation effect in all three of our missing bodies, rather than a linear relationship (R^2^ = 0.25).

### 2.7. Expression Changes in Homologous Chromosomes

**Figure 6:**
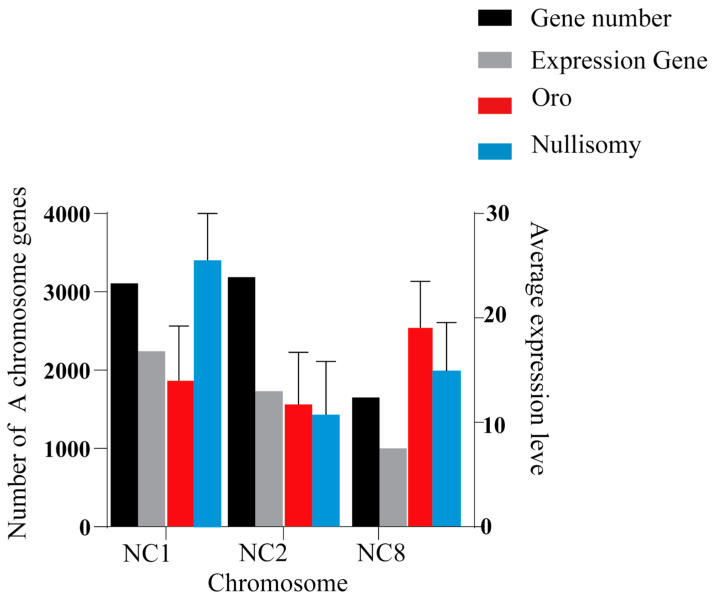
The expression level of homologous chromosomes. The number of homologous genes corresponding to the A chromosome and the number of expressions, as well as the average expression of homologous A genes. The red columns indicates the average expression level of Oro on chromosome A1, and the blue indicates the average expression level of the deficient body (NC1, NC2, and NC8) corresponding to chromosome A.

In *Brassica napus*, chromosome A and C exhibit high levels of homology. This raises the question of how chromosome A responds to the loss of homologous chromosome C, particularly in terms of enhancing or reducing dosage compensation. To elucidate the expression differences among various chromosomes, we compared the homologous genes of chromosomes A across C1, C2, and C8, and observed 3109, 3189, and 1651 homologous genes in NC1, NC2, and NC8, respectively (Figure 6). Notably, significant differences were observed in the gene expression ratio of 72.11% (2242), 54.31% (1732), and 60.57% (1000), respectively, as determined by a Student’s T-test (*p* < 0.05) (Figure 6). To assess changes in the expression of these homologous genes, we compared the average gene expression levels across the three homologous chromosomes, excluding genes that were not expressed in either euploidy or aneuploidy and retaining only those expressed in at least one strain. The results indicate that the average gene expression levels in NC2 and NC8 were lower than those in the euploid, while NC1 exhibited the opposite trend, with its average gene expression level being higher. These findings suggest the presence of distinct mechanisms of response to chromosome deletion in different aneuploidy.

### 2.8. Enrichment Analysis of DEGs

**Figure 7:**
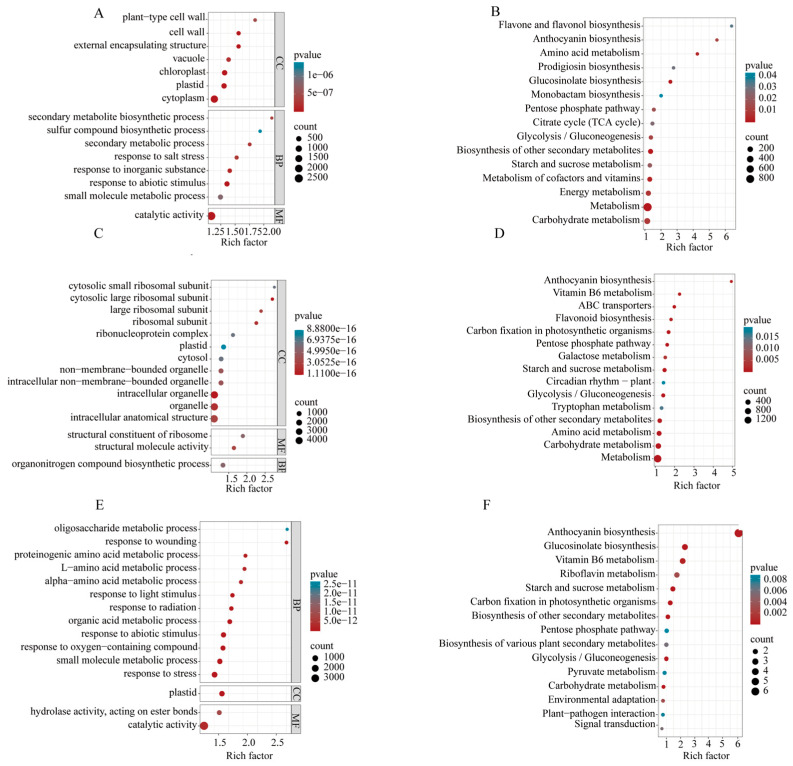
GO and KEGG enrichment analysis of DEGs. (**A**) NC1 vs. Oro GO enrichment analysis; (**B**) NC1 vs. Oro KEGG enrichment analysis; (**C**) NC2 vs. Oro GO enrichment analysis; (**D**) NC2 vs. Oro KEGG enrichment analysis; (**E**) NC8 vs. Oro GO enrichment analysis; (**F**) NC8 vs. Oro KEGG enrichment analysis. CC represents cell components, BP represents biological processes, and MF represents molecular functions.

The Goatools tool identifies GO annotations and predicts the function of DEGs by categorizing them into various biological processes. The selected enrichment pathways were all metabolic pathways with significant enrichment of DEGs, that is, the P-adjust *p*-value < 0.05. In the comparison of Ctr vs. NC1 DEGs are primarily enriched in biological processes related to metabolism, reaction to substances, and catalytic activity (Figure 7A). In the Ctr vs. NC2 comparison, DEGs are predominantly associated with ribosomal subunits and ribosomal proteins (Figure 7C). Additionally, in Ctr vs. NC8, DEGs are mainly enriched in responses to abiotic stimuli (Figure 7E). The KEGG enrichment analysis showed that the primary enrichment occurs in pathways related to amino acid metabolism in Ctr vs. NC1 anthocyanin synthesis, glycolysis, and other pathways (Figure 7B), while in the Ctr vs. NC2, the enrichment is primarily in metabolism, circadian regulation, and anthocyanin synthesis (Figure 7D). In Ctr vs. NC8, DEGs predominantly enriched in the pathways associated with carbon fixation, glycolysis, and signal transduction (Figure 7F). Therefore, plants will change the expression pattern of the remaining genes involved in biological processes in response to C chromosome deletion, thereby affecting the phenotype or even the physiological basis of the plant recovery and euploid maintenance.

In addition, in order to identify the occurrence of abnormal phenotypes due to the absence of C staining, we screened out the genes controlling different abnormal phenotypes in *Brassica napus*. According to previous studies, Liu screened 2127 SNP related to plant branching and growth in *Brassica napus* by genome-wide association (GWAS), and these genes were related to phosphorus content [28]. In the previous study, a total of 450 auxin-related genes were found in C2 deficiency [7]. Subsequently, the genes related to spiny development discovered in *Arabidopsis thaliana*, *Brassica oleracea*, and *Brassica nigra* were subjected to Blast with *Brassica napus*, and a total of 46 related genes were identified, including transcription factors such as *GL1*, *GL2*, *TRY, CPC*, and *EGL3* (Table A4). Most of these genes are concentrated in the pathways of energy metabolism, photosynthesis, and glucose metabolism.

## 3. Discussion

### 3.1. Effects of Aneuploidy on Phenotypic Variation and Function

Previous studies have demonstrated that aneuploidy leads to distinct phenotypic abnormalities in both plants and animals, which usually results in an imbalance in gene dose. While similar defects are observed across different aneuploidies, these variations are specific to the particular chromosome involved [29,30]. For instance, human individual lesions, due to the presence of extra chromosomes such as trisomy 13 (Patau syndrome), trisomy 18 (Edwards syndrome), trisomy 21 (Down syndrome), and sex chromosome trisomy (Klinefelter syndrome), all exhibit unique phenotypic characteristics, including intellectual disabilities and varying degrees of lethality. These differences highlight the complex effects of chromosomal imbalances on organismal development [31,32]. Aneuploidy in plants leads to significant phenotypic variation. For example, trisomic maize plants exhibits distinct changes in leaf and ear structure, overall plant morphology, and developmental stages [32]. Similarly, trisomic *Datura* strains show alterations in leaf and flower morphology [2]. Chromosome loss in plants frequently results in genomic instability, especially during mitosis, and is associated with diverse phenotypic changes, including growth retardation, altered disease resistance, and dwarfism. In *Orchidaceae*, chromosome loss can reduce fertility in several species, with some populations experiencing compromised biological functions due to incomplete genomes [33]. Crops like maize and wheat show more pronounced phenotypic changes under chromosomal loss, including impaired stem development, reduced seed size, and diminished stress resistance [18,34]. Research has indicated that plants with dropping chromosomes exhibit greater variability in response to environmental stress, potentially due to genomic heterogeneity [35]. In this study, we photographed the phenotypes of three deficient materials (NC, NC2, and NC8) and the euploid Oro at both the seedling and flowering stages. A previous study reported that when different copies of C2 chromosome were missing in *Brassica napus*, the flowering time was 10 days earlier than Oro, but C2 was absent about 3 months earlier, so we hypothesized that a similar time difference existed in NC2 [3]. Compared to Oro, the three deficient materials displayed more pronounced phenotypic traits. For instance, NC1 exhibited burr growth at the seedling stage, NC2 flowered approximately two months earlier, and NC8 produced more branches. These phenotypic differences likely result from an imbalance in gene copy numbers, with specific “key” genes contributing to these effects. The abnormal phenotypes are caused by disruptions in the regulation of gene expression. We identified 2218, 4945, and 5003 newly expressed genes, as well as 3597, 5302, and 4148 silenced genes in the three missing chromosomal bodies, respectively. The appearance of these newly expressed and silenced genes plays a crucial role in the manifestation of abnormal phenotypes, such as burr formation, early flowering, and increased branching. For example, a substantial number of genes regulating flowering time were identified in C2-deficient plants [7]. These same genes also appear on the missing chromosomes in our study and are involved in leaf burr development.

Different chromosomes contribute differently to plant growth, development, environmental adaptability, and biological evolution, with these differences regulated by gene dosage compensation and chromosomal rearrangement mechanisms [36]. Gene expression across subgenomes may vary under different environmental conditions. In *Brassica napus*, the two subgenomes contribute differently to plant growth, development, and stress resistance. For instance, the A genome plays a dominant role in stress resistance, while the C genome primarily regulates morphological changes [37,38]. When chromosome dosage becomes unbalanced, gene expression regulation is disrupted. For instance, the integration of different C chromosomes to radish resulted in diverse phenotypic outcomes, including variations in plant height, leaf morphology, and flowering time. Meanwhile, agronomic traits such as growth rate and disease resistance were also modulated through gene regulation [39]. Similarly, the introduction of B genome chromosomes from *Brassica nigra* significantly altered the transcriptional landscape of the host C genome, leading to the widespread upregulation or downregulation of host genes. These changes affected multiple functional categories, including metabolism, signal transduction, and stress responses [40]. These results highlight the influence of chromosome copy number changes on the tendency of abnormal expression in plants.

### 3.2. Global Perturbation of Gene Expression

Transcriptional changes resulting from aneuploidy should be analyzed based on alterations in chromosome or chromosome fragment numbers, as well as the occurrence of these changes in cis- or trans-regions. This distinction helps determine whether transcription or protein expression changes are proportional to DNA copy number variations or if the cell mitigates aneuploidy effects through dose compensation [7]. While gene expression often correlates with copy number, studies on aneuploid *Arabidopsis* revealed that chromosomal abnormalities can upregulate or downregulate multiple genes. However, these changes are not always directly linked to chromosome copy number, indicating complex regulatory mechanisms beyond simple dosage effects [14]. Furthermore, research on maize has demonstrate that aneuploidy can exert effects on gene expression across various tissues, resulting in altered tissue-specific expression patterns [32]. While gene expression has been extensively studied in yeast and mammals, findings diverge in fruit flies and plants, suggesting the existence of diverse mechanisms to counteract aneuploidy-induced gene dose imbalances. These mechanisms serve to prevent both overexpression and underexpression due to abnormal chromosome numbers. While the specific pathways vary among species, most organisms achieve compensation by regulating gene expression on affected chromosomes. Comparisons between aneuploid and normal diploid cells show disproportionate changes in certain gene expression levels. For instance, some genes are suppressed to prevent overexpression when chromosome numbers increase, while others are upregulated to compensate for reduced gene dosage. These findings highlight the complexity and species-specific nature of dose-compensation mechanisms in maintaining cellular homeostasis in the presence of chromosomal imbalance.

Using RNA-seq, we traced the origin of aneuploid deletion chromosomes in *Brassica napus* and examined the significant impact of C chromosome deletions on global gene expression. Abnormal phenotypes were observed in the three losing chromosomes, likely due to the specific function performed on the C homologous gene. Some regulatory interactions and gene networks associated with the C gene may be compensated by alternative mechanisms. To further investigate the function of the C homologous chromosome, we analyzed the expression of the C homologous gene. In three kinds of nullisomy, the deletion of C1, C2, and C8 resulted in the expression of 68 new genes in A1, while 46 and 53 new genes were expressed in A2 and A8, respectively. These newly expressed genes may contribute to the observed phenotypic changes. When the C chromosome is completely deleted, its independent function in regulating homologous genes suggests that the losing genome’s integrity can be partially restored. This supports the genomic balance hypothesis, which distinguishes chromosomal variation from ploidy changes, as a valid explanation for our findings [41]. It is conceivable that a large number of homologous genes independently contribute to the biological function and viability of the deficient chromosome.

### 3.3. Compensatory Upregulation of Chromosomes

Dose-compensation mechanisms are critical evolutionary strategies enabling organisms to adapt to chromosomal number changes. In aneuploidy (e.g., trisomy or monosomy), gene expression may become dysregulated, disrupting cellular and organismal functions. To counterbalance this, organisms activate diverse compensation mechanisms across multiple regulatory levels, including gene expression, transcription, and epigenetics. Key processes such as gene dose compensation, epigenetic modifications, and transcriptional regulation help stabilize the genome and mitigate harmful effects. Although the adaptability of these mechanisms differs between plants and animals, most organisms restore genomic function through the feedback regulation of gene expression, thereby ensuring survival and stability [12,33,42].

**Figure 8:**
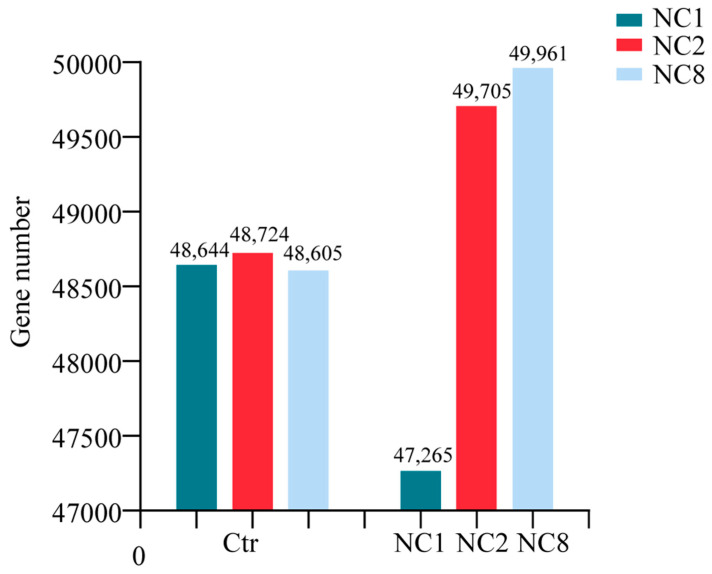
Expressed genes from the remaining chromosomes of the aneuploid plants. Number of remaining chromosome gene expressions after the deletion of the chromosome.

To explore the compensation mechanism in *Brassica napus* following the deletion of C chromosomes, we analyzed gene expression files on the remaining chromosomes. Our findings indicate that C2 loss triggers the compensatory upregulation of specific genes in the remaining chromosome during aneuploidy, which was in line with the previous report [7]. This phenomenon was also observed in NC8. Conversely, in NC1, the C1 chromosome resulted in a decrease in gene expression across the remaining chromosomes, which was unexpected. As shown in Figure (Figure 5A), the average expression level of chromosome A1 in NC1 was significantly higher than that in Oro (*t*-test *p* < 0.01), contrasting with the results for NC2 and NC8 (Figure 6). Additionally, gene expression levels in NC2 and NC8 (49,705 > 48,724 χ^2^ *p* < 0.05, 49,961 > 48,605 χ^2^ *p* < 0.01) were higher than those in Oro (Figure 8). However, the average expression levels of chromosomes A2 and A8 were lower in NC2 and NC8 compared to Oro. Furthermore, Makarevitch observed a different compensation mechanism in *Zea mays*, in which gene expression is regulated through buffering the gene dosage, particularly in specific chromosomal regions or genes, and it has also been observed in *Drosophila melanogaster* [32,43]. In the case of whole-chromosome aneuploidy, compensation mainly occurs through direct gene dosage regulation. In contrast, partial aneuploidies may modulate gene expression via local regulatory networks, thereby mitigating the impacts of dose imbalance. In Drosophila, compensatory responses to aneuploidy include buffering, feedback, and feedforward, which may function independently or in combination [43,44,45]. Buffering is the passive absorption of gene dose perturbations by the system’s inherent properties, maintaining biochemical balance. The loss of a gene copy, such as through reduced gene expression, can lead to elevated steady-state mRNA levels, contributing to compensation. Feedback serves as an error control mechanism that identifies deviations from a normative state and initiates corrective actions, whereas feedforward predicts potential impacts of perturbations on the system [44,46].

Thus, the creation of nullisomy materials for *Brassica napus* can provide new germplasm resources for the genetic and breeding studies of *Brassica napus*, while also simplifying the complexity of *Brassica napus* genome, thereby facilitating the interaction and evolution of A and C genomes of *Brassica* genus.

## 4. Materials and Methods

### 4.1. Plant Materials

*Brassica napus* L. Oro was hybridized with exfoliated Chinese cabbage (RBR Oro) to obtain sesquid diploid hybrid F1, which was backcrossed with the maternal plant Oro to obtain BC1F1, and then the young ovary was selected, the somatic cells were counted, and the plants with fewer than 38 chromosomes were artificially pollinated to obtain substitute plants. These plants were initially screened for molecular markers and then FISH analysis was performed to determine the amount of lost staining. The materials used in this study were all planted in School of Life Sciences, Guizhou Normal University, Guiyang, China (26°23′11″ N, 106°38′32″ E).

### 4.2. Cytological Analysis and Fluorescence In Situ Hybridization

To determine the number of chromosomes in the plants, we collected the young buds, removed the ovaries, and treated them with 2 mM 8-hydroxyquinoline solution under dark conditions for 3–4 h. Subsequently, the ovaries were transferred to Carnoy fixative (ethanol: acetic acid volume ratio 3:1) and fixed overnight. Following fixation, the ovaries were placed in a water bath containing 1 M HCl at 60 °C for 8 min. After this treatment, the ovaries were transferred to distilled water and soaked for 1 min [47]. The ovaries were gently placed on a slide, mused with tweezers, and stained with a modified Carbosin fuchsin solution (Nikon, Tokyo, Japan) for 3 min.

In order to detect the genome structure and chromosome behavior of the plant, fluorescent hybridization was performed using a C genome-specific primer repeat sequence as the probe. According to the manufacturer’s protocol, biotin 11-dUTP random primer labeling of C-genome-specific BAC BoB014006 plasmid DNA in Brassica was performed using the BioPrime DNA Labeling System kit (Beyotime, Shanghai, China). The slide preparation method adhered to the procedure established by Zhong, while the FISH operational protocol followed that of Cui, with only minor differences [48,49]. Specifically, prior to the second round of FISH, the water bath temperature was reduced to 40 °C and formamide concentration was set at 20%. The FISH results were observed using a fluorescence microscope coupled with a CCD (Charge Couple Device) camera (Ni80, Nikon, Tokyo, Japan). Adobe Photoshop (version 2024) was employed to adjust the background to black and to modify the contrast and exposure.

### 4.3. RNA Extraction and cDNA Library Preparation

The plants grown in the growth chamber were labeled according to their growth sequence, and the third set of petioleless leaves newly developed from three strains of each genotype were collected and divided into three replicates. The leaves of three aneuploid lines (NC1, NC2, and NC8) and euploid Oro with similar growth cycles and growth trends were collected with three biological replicates each, totaling 12 samples, 0.1–0.2 g per sample. These leaves were immediately stored in liquid nitrogen for RNA extraction. Total RNA was extracted from each genotype using TRIZol (Invitrogen, Life Technologies China) in accordance with the manufacturer’s protocol. The concentration and purity of total RNA were measured using Nanodrop2000 (Thermo Scientific, Shanghai, China), and RNA integrity was further measured through 1.5% agarose gel electrophoresis. Total RNA was enriched with Oligo (dT) and subsequently fragmented into small fragments with the addition of fragmentation buffer. Then, the first strand of cDNA was synthesized using reverse transcriptase (Thermos Scientific RevertAid premix, Shanghai, China) followed by the synthesis of the second strand through the addition of End Repair Mix (Thermo Scientific Rapid DNA end repair kit) to create blunt ends. An adenine base was then added to the three ends to facilitate the subsequent attachment of the adapter sequence. The products linked to the adapter were purified and sorted, after which PCR amplification was performed with the sorted products to purify the library. Subsequent transcriptome sequencing was performed at Shanghai Majorbio Company (Shanghai, China).

### 4.4. Differential Gene Enrichment Analysis

We used DESeq2 (version 1.48.0)software [50] to identify DEGs in order to ensure that the selected differential genes have statistical significance in biology, avoid picking out some small but insignificant changes, and reduce the interference of false positives, and in order to achieve a high success rate of subsequent qRT-PCR verification, we applied thresholds of false discovery rate (FDR) < 0.05 and |Log2 fold-change (FC)| ≥ 1. For the identified DEGs, gene ontology (GO) and Kyoto Encyclopedia of Genes and Genomes (KEGG) enrichment analyses were conducted using the clusterProfiler package (version 4.4.1) [51] and the KEGG database [52]. Multiple testing correction methods, including Bonferroni, Holm, Sidak, and FDR, were applied to adjust the *p*-values, with a corrected *p* < 0.05 deemed significantly enriched in GO and KEGG analyses. We ensured the quality of our data by removing low-quality sequences, connectors, and contaminated or overexpressed sequences to yield clean reads, which were then compared against the reference genome (*Brassica napus* annotation v0. gff3). Finally, an enrichment analysis was carried out using Goatools [53], with the Fisher exact test employed, and *p*-values corrected using various testing methods. The KEGG pathway enrichment analysis was conducted using the Python (version 3.11.0) scipy software package, following the same calculation principles as the GO functional enrichment analysis, with calculations executed via Fisher’s exact tests. Gene expression data can be queried through NCBI numbers, NC1 and Oro (GSE180585), Oro, and NC2 and NC8 (PRJNA1195237). Samples PRJWT51-PRJWT53 and O-1–O-3 stand for Oro, C-1–C-3 stand for NC1, PRJCD51–PRJCD53 stand for NC2, and PRJBC51–PRJBC53 stand for NC8.

### 4.5. RT-qPCR Analysis

To verify the reliability of the transcriptome, we extracted the total RNA from the three nullisomy lines and one euploid Oro. The RNA samples used for the qRT-PCR assay were the same as those employed in the RNA-sequencing experiment. cDNA was synthesized under the action (*Actin-7*) of reverse transcriptase, and primers were designed with Primer 6.0 based on the reference genes (Table A1). The qRT-PCR assay was performed using SYBRPRIME qPCR (Fast HS) kit.

## 5. Conclusions

In this study, we analyze the changes in gene expression patterns after the deletion of C chromosome pairs (NC1, NC2, and NC8) in three rapeseed species by RNA-seq technology and discuss the compensation mechanism caused by the deletion of these three C chromosomes to reveal how aneuploidy responds to partial chromosome loss at the transcriptional level. We found that there were different abnormal phenotypes and different compensation mechanisms due to chromosome loss in the three types of *Brassica napus* nullisomy lines. Our findings underscore that plants employ different compensation mechanisms to cope with the loss of different chromosomes. By comparing the phenotypic observations and molecular characteristics of different aneuploid materials, the target genes or quantitative trait loci (QTLs) can be precisely mapped; meanwhile, the nullisomy material is also an important material source in breeding. This is critical for elucidating the genetic basis of complex traits such as stress resistance, yield, and quality. Moreover, various aneuploid lines serve as ideal models for investigating genome stability and chromosome behavior.

## Figures and Tables

**Table 1:** Genes are differentially expressed along the remaining chromosomes in different multiples.

	Oro_vs_NC1					Oro_vs_NC2					Oro_vs_NC8				
	UP	Ratio (%)	Down	Ratio (%)	Total	UP	Ratio (%)	Down	Ratio (%)	Total	UP	Ratio (%)	Down	Ratio (%)	Total
DEGs	3253	51.41	3043	48.59	6296	6314	55.11	5144	44.89	11,458	4967	53.24	4363	46.76	9330
FC ≥ 10	921	49.84	927	50.16	1848	1932	61.31	1219	38.69	3151	1259	56.11	985	43.89	2244
FC ≥ 100	368	55.76	292	44.24	660	587	58.41	418	41.59	1005	269	44.98	329	55.02	598
FC ≥ 1000	46	63.01	27	36.99	73	110	62.15	67	37.85	177	32	33.68	63	66.32	95

**Table 2:** Proportion of expressed genes (EGs) and DEGs along all *Brassica napus* Ctr vs. NC1.

Chromosome	Number of Reference Genes	Expressed Genes	R (%) (EGs/RGs)	DEGs	R (%) (DEGs/EGs)	Group
A01	4296	2350	54.70	824	35.06	High
A02	4203	2066	49.17	301	14.57	High
A03	5946	3210	53.99	421	13.12	Middle
A04	3014	1527	50.66	220	14.41	High
A05	5002	2007	40.12	256	12.76	Middle
A06	4533	2388	52.68	339	14.20	High
A07	3904	2185	55.97	185	8.47	Middle
A08	3222	1732	53.76	150	8.66	Middle
A09	7218	3479	48.20	393	11.30	Middle
A10	3021	1748	57.86	121	6.92	Middle
A	44,359	22,692	51.16	3210	14.15	—
C01	5168	1509	29.20	827	54.80	—
C02	5574	2218	39.79	334	15.06	High
C03	8178	4067	49.73	232	5.70	Low
C04	6183	2841	45.95	195	6.86	Middle
C05	5761	2922	50.72	160	5.48	Low
C06	4597	2270	49.38	125	5.51	Low
C07	5507	2683	46.72	151	5.63	Low
C08	5349	2548	47.64	131	5.14	Low
C09	6245	2607	41.75	226	8.67	Middle
C	52,562	23,665	45.02	2381	10.06	—
Total	96,924	46,357	47.83	5591	12.03	—

Number of reference genes represents all the genes present in the chromosome, RGs; expressed genes indicates the genes expressed in the chromosome, that is, the genes with TPM > 0.

**Figure A3:**
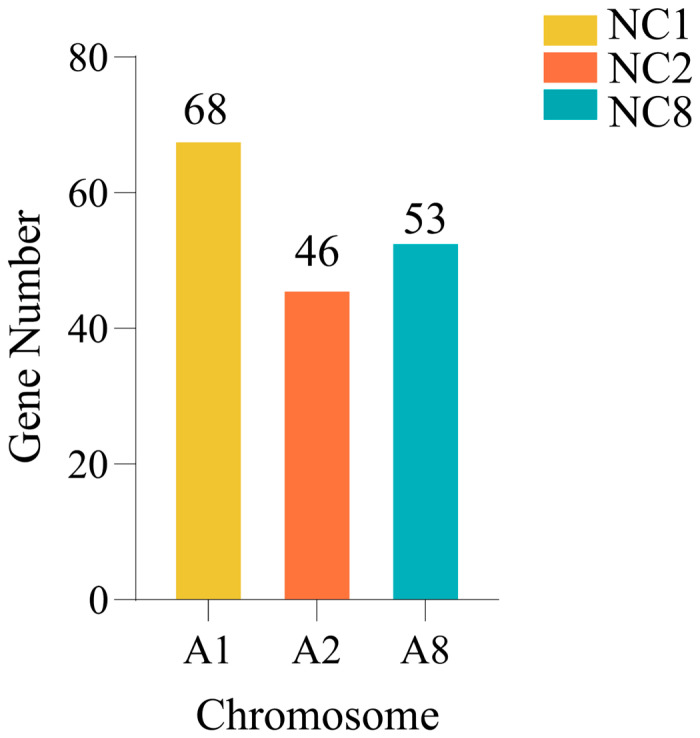
The number of newly expressed genes on the homologous A chromosome corresponding to the three nullisomy.

## Data Availability

Gene expression data can be queried through NCBI numbers; NC1 (GSE180585), NC2 and NC8 (PRJNA1195237).

## Appendix A

**Table A1:** Primer sequence of internal reference actin7 for qRT-PCR analysis.

Genome ID	Name	Forward Primer (5′-3′)	Reverse Primer (5′-3′)
BnaC02g00690D	Actin-7	GGTTCGACCATGTTCCCAGGT	GTGCTGAGGGATGCAAGGATG

**Table A2:** Proportion of expressed genes (EGs) and DEGs along all *Brassica napus* Ctr vs. NC2 chromosomes.

Chromosome	Number of Reference Genes	Expressed Genes	R (%) (EGs/RGs)	DEGs	R (%) (DEGs/EGs)	Group
A01	4296	2306	53.68	437	18.95	Middle
A02	4203	2121	50.46	338	15.94	Low
A03	5946	3398	57.15	546	16.07	Low
A04	3014	1579	52.39	363	22.99	High
A05	5002	2274	45.46	555	24.41	High
A06	4533	2496	55.06	457	18.31	Middle
A07	3904	2235	57.25	466	18.31	Middle
A08	3222	1824	46.61	332	18.20	Middle
A09	7218	3616	50.10	811	22.43	High
A10	3021	1762	58.33	359	6.92	Low
A	44,359	23,611	53.23	4664	19.75	—
C01	5168	2527	48.90	540	21.37	Middle
C02	5574	1061	19.03	817	77.00	—
C03	8178	4124	50.43	834	20.22	Middle
C04	6183	2758	44.61	631	22.88	High
C05	5761	2951	51.22	570	19.32	Middle
C06	4597	2325	50.58	380	16.34	Low
C07	5507	2732	49.61	436	15.96	Low
C08	5349	2576	48.16	466	18.09	Middle
C09	6245	2734	43.78	585	21.40	High
C	52,562	23,788	45.26	5259	22.11	—
Total	96,924	47,399	48.90	9921	20.93	—

Number of reference genes represents all the genes present in the chromosome, RGs; expressed genes indicates the genes expressed in the chromosome, that is, the genes with TPM > 0.

**Table A3:** Proportion of expressed genes (EGs) and DEGs along all *Brassica napus* Ctr vs. NC8 chromosomes.

Chromosome	Number of Reference Genes	Expressed Genes	R (%) (EGs/RGs)	DEGs	R (%) (DEGs/EGs)	Group
A01	4296	2351	54.73	323	13.74	Low
A02	4203	2138	50.87	450	21.05	High
A03	5946	3373	56.73	627	18.59	Middle
A04	3014	1581	52.46	379	23.97	High
A05	5002	2305	46.08	567	24.60	High
A06	4533	2499	55.13	509	20.37	High
A07	3904	2275	58.28	300	13.19	Low
A08	3222	1832	56.86	293	15.99	Middle
A09	7218	3639	50.42	622	17.09	Middle
A10	3021	1820	60.24	194	10.66	Low
A	44,359	23,813	53.68	4264	17.91	—
C01	5168	2569	49.71	361	14.05	Middle
C02	5574	2459	44.12	368	14.97	Middle
C03	8178	4188	51.21	586	13.99	Middle
C04	6183	2751	44.49	563	20.47	High
C05	5761	3019	52.40	424	14.04	Middle
C06	4597	2348	51.08	325	13.84	Middle
C07	5507	2781	50.50	382	13.74	Low
C08	5349	1093	20.43	918	83.99	—
C09	6245	2805	44.92	362	12.91	Low
C	52,562	24,013	45.69	4289	17.86	—
Total	96,924	47,826	49.34	8553	17.88	—

Number of reference genes represents all the genes present in the chromosome, RGs; expressed genes indicates the genes expressed in the chromosome, that is, the genes with TPM > 0.

**Table A4:** Gene expression of control plant spine development.

Genes	Gene ID	Gene Expression (TPM)	
		Oro	NC1
*ARF*	BnaA01G0267900ZS	8.94	13.08
	BnaA02G0396100ZS	2.82	2.51
	BnaA02G0396600ZS	4.93	4.83
	BnaA04G0008800ZS	6.74	3.94
	BnaA05G0014400ZS	0.08	0.12
	BnaA09G0077100ZS	21.69	16.17
	BnaA09G0559300ZS	4.19	1.90
	BnaA09G0702900ZS	3.54	2.14
	BnaC01G0174100ZS	0.37	0.27
	BnaC01G0328100ZS	7.26	1.99
	BnaC02G0529200ZS	11.68	8.16
	BnaC03G0559800ZS	21.86	17.08
	BnaC04G0014300ZS	0.45	0.10
	BnaC04G0265800ZS	3.71	0.77
	BnaC08G0407500ZS	4.69	2.74
	BnaC09G0068500ZS	3.51	2.52
*TRY*	BnaA02G0140700ZS	0.00	0.00
	BnaA03G0131600ZS	1.59	0.00
	BnaA10G0097300ZS	0.00	1.57
	BnaC01G0222200ZS	1.66	0.00
	BnaC01G0222400ZS	3.82	0.00
	BnaC02G0178400ZS	1.46	0.00
	BnaC03G0153000ZS	0.10	1.98
	BnaC09G0353000ZS	2.30	0.60
*R2R3*	BnaA01G0097700ZS	0.00	0.00
	BnaA08G0111700ZS	0.00	0.00
	BnaC01G0119400ZS	0.00	0.00
	BnaC03G0706500ZS	0.00	0.02
	BnaC07G0425500ZS	0.00	0.00
*EGL3*	BnaA09G0134400ZS	0.32	0.41
	BnaA09G0148900ZS	0.90	0.15
	BnaC09G0142800ZS	0.67	0.72
	BnaC09G0162100ZS	0.81	0.21
*WU*	BnaA06G0314000ZS	0.00	0.00
	BnaA09G0114500ZS	0.00	0.00
	BnaC03G0508400ZS	0.00	0.00
	BnaC09G0117600ZS	0.00	0.00
*MYB3*	BnaA07G0121400ZS	4.52	2.11
	BnaC07G0178800ZS	2.86	2.14
*CPC*	BnaA04G0292300ZS	0.00	0.00
	BnaC04G0015600ZS	0.00	0.00
*GL1*	BnaA09G0040500ZS	0.00	0.00
	BnaC07G0310800ZS	0.00	0.00
*TMM*	BnaA07G0224900ZS	1.02	0.12
	BnaC06G0242000ZS	1.63	0.1
*GL2*	BnaC06G0240800ZS	0.08	0.12
